# The expression of *MDM2/CDK4* gene product in the differential diagnosis of well differentiated liposarcoma and large deep-seated lipoma

**DOI:** 10.1054/bjoc.1999.1090

**Published:** 2000-03-06

**Authors:** S Pilotti, G Della Torre, A Mezzelani, E Tamborini, A Azzarelli, G Sozzi, M A Pierotti

**Affiliations:** Departments of Pathology and Cytopathology, Experimental Oncology, Surgery of Soft Tissue Tumors, Istituto Nazionale per lo Studio e la Cura dei Tumori, Via G. Venezian, Milano, 1–20133, Italy

**Keywords:** MDM2, CDK4, lipoma, liposarcoma lipoma-like, differential diagnosis

## Abstract

Ordinary lipomas are cytogenetically characterized by a variety of balanced rearrangements involving chromosome segment 12q13–15, whereas well differentiated liposarcomas (WDL) show supernumerary ring and giant marker chromosomes, known to contain amplified 12q sequences. The tight correlation between the presence of ring chromosomes and both amplification and overexpression of *MDM2* and *CDK4* genes suggests the exploration of the possibility that immunocytochemistry (ICC) might assist in the differential diagnosis of lipoma-like well differentiated liposarcomas (LL-WDL) and large deep-seated lipomas (LDSL). For this purpose, 21 cases of the former and 19 cases of the latter tumours were analysed by ICC and, according to the availability of material, by molecular and cytogenetic approaches. All lipomas displayed a null MDM2/CDK4 phenotype, whereas all LL-WDL showed MDM2/CDK4 or CDK4 phenotypes. Southern blot analysis performed on 16 suitable cases, complemented by fluorescence in situ hybridization and classical cytogenetic analysis in 11 cases, was consistent with, and further supported the immunophenotyping data. In conclusion, *MDM2/CDK4* product-based immunophenotyping appears to represent a valuable method for the categorization of arguable LDSL. © 2000 Cancer Research Campaign


*British Journal of Cancer* (2000) **82**(7), 1271–1275
© 2000 Cancer Research Campaign
DOI: 10.1054/ bjoc.1999.1090, available online at http://www.idealibrary.com on IDEAL®

### The expression of *MDM2/CDK4* gene product in the differential diagnosis of well differentiated liposarcoma and large deep-seated lipoma

**S Pilotti[1], G Della Torre[2], A Mezzelani[1], E Tamborini[1], A Azzarelli[3], G Sozzi[2] and MA Pierotti[2]**

Departments of [1]Pathology and Cytopathology, [2]Experimental Oncology and [3]Surgery of Soft Tissue Tumors, Istituto Nazionale per lo Studio e la Cura dei Tumori, Via G. Venezian, 1–20133 Milano, Italy

**Summary** Ordinary lipomas are cytogenetically characterized by a variety of balanced rearrangements involving chromosome segment 12q13–15, whereas well differentiated liposarcomas (WDL) show supernumerary ring and giant marker chromosomes, known to contain amplified 12q sequences. The tight correlation between the presence of ring chromosomes and both amplification and overexpression of *MDM2* and *CDK4* genes suggests the exploration of the possibility that immunocytochemistry (ICC) might assist in the differential diagnosis of lipoma-like well differentiated liposarcomas (LL-WDL) and large deep-seated lipomas (LDSL). For this purpose, 21 cases of the former and 19 cases of the latter tumours were analysed by ICC and, according to the availability of material, by molecular and cytogenetic approaches. All lipomas displayed a null MDM2/CDK4 phenotype, whereas all LL-WDL showed MDM2/CDK4 or CDK4 phenotypes. Southern blot analysis performed on 16 suitable cases, complemented by fluorescence in situ hybridization and classical cytogenetic analysis in 11 cases, was consistent with, and further supported the immunophenotyping data. In conclusion, *MDM2/CDK4* product-based immunophenotyping appears to represent a valuable method for the categorization of arguable LDSL. © 2000 Cancer Research Campaign

**Keywords:** MDM2; CDK4; lipoma; liposarcoma lipoma-like; differential diagnosis

Non-subcutaneous ordinary lipomas, including subfascial and intramuscular lipomas, account for 40% of ordinary lipomas (Fletcher et al, 1996). These tumours, also called deep-seated ordinary lipomas, may achieve a large size, particularly the intramuscular ones. In these cases the differential diagnosis between large deep-seated ordinary lipoma (LDSL) and the lipoma-like subtype of well differentiated liposarcoma (LL-WDL) may be difficult due to their similar histologic characteristics and focality of nuclear atypia (Rosai et al, 1996).

Furthermore, although lipomatous tumours in the retroperitoneum are mainly represented by well differentiated liposarcomas (WDL), lipomas may also appear at this site (DeWeerd et al, 1952). Thus, it would be valuable to support histologic classification with a simple immunophenotypic procedure in arguable cases. The 12q13–15 chromosome region is complex and contains several genes that are amplified or rearranged in lipomatous tumours such as the *MDM2*, *CDK4*, *SAS* or *CHOP* and *HMGI-C* (High Mobility Group I-C protein) genes respectively (Smith et al, 1992; Forus et al, 1993; Khatib et al, 1993; Leach et al, 1993; Schoenmakers et al, 1995). *HMGI-C* gene translocations have been suggested to facilitate the development of lipomas (Schoenmakers et al, 1995; Tkachenko et al, 1997). On the other hand, *MDM2* and *CDK4* amplification and overexpression, which in turn correlate with the presence of chromosomes containing material from 12q, have been indicated as frequent in WDL (Dal Cin et al, 1993; Khatib et al, 1993; Pedeutour et al, 1993; Hunter and Pines, 1994; Nilbert et al, 1994; Pilotti et al, 1997, 1998). More recently it has also been shown that overexpression of 12q sequences is a recurrent finding in WDL but not in lipoma (Mandahl et al, 1996; Szymanska et al, 1997).

Here we applied a *MDM2/CDK4* gene-product-based immunophenotypic analysis, complemented in suitable cases by molecular and cytogenetic approaches, to a series of LL-WDLs and of LDSLs for comparison purposes.

The results show that MDM2/CDK4 immunophenotyping may assist in the differential diagnosis of the two entities.

*Received 16 July 1999*
*Revised 16 November 1999*
*Accepted 24 November 1999*

*Correspondence to:* S Pilotti

### MATERIALS AND METHODS

### Materials

The case material consisted of 21 cases of LL-WDL including 12 primary and nine recurrent tumours and 19 primary LDSL from patients treated at our institution between 1988 and 1997. Eleven cases had been included in a previously reported series (cases 5, 9–13, 17–21) (Pilotti et al, 1997). All cases have been reviewed in order to verify the diagnosis and select representative tumoural samples for immunophenotypic analysis. The morphologic diagnoses were confirmed in all cases following the criteria of the pathologists of the CHAMP study group (Willen et al, 1998) and the criteria of Evans (1988) updated by Mentzel and Fletcher (1995). The cases were consecutive according to the criteria of selection: histologic subtype (LL-WDL) for liposarcoma and size (> 7 cm in largest diameter) for lipoma.

The age and sex of the patients as well as the location, type of specimens analysed (primary, recurrence) and size of the tumours are presented in Tables 2 (LL-WDL) and 3 (LDSL).

Table 1 Lipoma-like well differentiated liposarcomas: clinical features

| Case no. | Age/Gender | Site | Primary treatment | Number/Time of local recurrence | Treatment of recurrence | Follow-up |
|---|---|---|---|---|---|---|
| 1 | 64/F | Thorax wall | Resection | 2, at 7 and 9 years | Resection + CT | NED, 1 year |
| 2 | 84/M | Thigh | Resection | – | – | NED, 1 year |
| 3 | 42/M | Retroperitoneum | Resection | – | – | Lost |
| 4 | 36/F | Retroperitoneum | Resection[b] | 1, at 1 year | Resection + RT | AWD, 2 years |
| 5 | 49/M | Retroperitoneum | Resection[b] | 3, at 4, 6 and 9 years | Resection | AWD, 9 years |
| 6 | 50/M | Retroperitoneum | RT[a] resection[b,c] | 1, at 1 year | RT + resection | NED, 2 years |
| 7 | 59/M | Thigh | Resection[b] | 1, at 4 years | Resection + CT | NED, 1.5 years |
| 8 | 67/M | Retroperitoneum | Resection[b] | 1, at 3 years | Resection | NED, 5 years |
| 9 | 56/F | Retroperitoneum | Resection | – | – | NED, 4 years |
| 10 | 57/F | Retroperitoneum | Resection[b] | 1, at 3 years | Resection | lost |
| 11 | 64/M | Retroperitoneum | CT[a] resection[b] | 2, at 2 and 5 years | Resection + RT | AWD, 5 years |
| 12 | 46/F | Retroperitoneum | Resection[b] | 2, at 3 and 6 years | Resection + CT + RT | AWD, 9 years |
| 13 | 51/F | Retroperitoneum | Resection[b] | 1, at 3 years | Resection | NED, 6 years |
| 14 | 62/M | Thigh | Resection | – | – | NED, 2 years |
| 15 | 72/M | Thigh | Resection | 2, at 4 and 7 years | Resection | NED, 9 years |
| 16 | 52/M | Shoulder | Resection | – | – | lost |
| 17 | 66/M | Thigh | Resection | 3, at 5, 12 and 13 years | Resection + CT | AWD, 2, 5 years |
| 18 | 45/F | Arm | Resection | – | – | NED, 1, 5 years |
| 19 | 28/M | Thigh | Resection | – | – | NED, 9 years |
| 20 | 26/F | Buttock | Resection | – | – | NED, 9 years |
| 21 | 44/F | Pelvis | Resection | 1, at 1 years | CT | lost |

RT = radiotherapy; CT = chemotherapy; NED = no evidence of disease; AWD = alive with disease. [a]For details see text. [b]Primary tumour resection performed elsewhere. [c]Synchronous renal carcinoma.

Histologically, the lipoma-like component constituted 100% of the tumour in 16 out of 21 LL-WDLS including primaries and recurrences. In three retroperitoneal cases a sclerosing subtype component constituted from 20% (case no. 3) to 30% (cases 4 and 5) of the total of sampled tumours respectively, and in two non-retroperitoneal tumours (cases 1 and 7) this component corresponded to less than 20%. In two cases radiotherapy (case no. 6) and chemotherapy (case no. 11) were performed before surgical resection of tumour samples for study.

### Immunocytochemistry, karyotyping and Southern blot analysis

Immunocytochemistry, karyotyping and Southern blot analysis were carried out as previously described (Pilotti et al, 1997, 1998). For immunostaining analysis from 2–8 LL-WDLs samples (for details see Table 2) and all samples of LDSLs were processed.

Suitable material for molecular analysis was available in nine out of 21 (42.8%) LL-WDLs and in seven out of 19 (36.8%) LDSLs. The material suitable for classic cytogenetic analysis accounted for three out of 21 and two out of 19 cases in the two groups respectively.

For Southern blotting, DNA was digested with the restriction enzyme *Kpn*I. Radiolabelled probes were: a 258-nucleotide *MDM2* genomic fragment amplified by PCR with primers within exons 2 and 3 of the gene (Zauberman et al, 1995), a 534-nucleotide *CDK4* genomic fragment generated by polymerase chain reaction (PCR) using the primers described by Khatib et al. (1993), and a PCR-generated *TP53* genomic fragment spanning nucleotides 13040–13450 of the gene. Hybridization with each probe resulted in a single band, which was of about 23 kb for the *MDM2* gene, 1.5 kb for the *CDK4* gene, and about 12 kb for TP53.

### Fluorescence in situ hybridization

Interphase fluorescence in situ hybridization (FISH) analysis was carried out on cryopreserved cytologic scrapings of the surgical specimen. Each slide was hybridized with the biotinylated probe Amplification unit probe 12q13 (Li Biomedical Corporation) corresponding to the amplicon *MDM2*, *SAS* and *GLI* genes. Hybridization was performed according to Lichter et al (1990), Mezzelani et al (1996) and the manufacturer's recommendations. The sample was scored as amplified when the single nuclei showed more than two signals. Metaphase FISH analysis was performed using whole chromosome 12 and 6 paintings (CAMBIO, Cambridge, UK) using standard procedures (Pilotti et al, 1997, 1998). Overall, cytogenetic analysis and/or interphase and metaphase FISH were performed in eight out of 21 cases of LL-WDL and in six out of 19 cases of LDSL.

### RESULTS

Table 1 summarizes the main clinical data, treatment and outcome on LL-WDLs. Thirteen out of 21 (61.9%) patients experienced from one to three recurrences, whereas only one case of recurrence was observed among the LDSLs (case no. 33). Given the uneventful follow-up of the remaining 18 cases a dedicated table has not been produced.

Immunophenotyping (Table 2) shows MDM2/CDK4 expression in 18 of the 21 cases (85.7%) and CDK4 immunoreactivity in 100% of the cases. The analysis of two samples was sufficient to achieve information about the immunophenotypic profile in the majority of the cases. In the three MDM2-CDK4+ cases (Table 2, cases 9, 14 and 16) all available samples were immunostained in order to confirm the null MDM2 immunophenotype.

All LDSL samples analysed turned out to be MDM2-CDK4- (Table 3). Tables 2 and 3 and Figure 1A–F summarize

© 2000 Cancer Research Campaign

**Table 2** Lipoma-like well differentiated liposarcomas: immunophenotyping, molecular and cytogenetic features (21 cases

| Case no. | Specimens analysed: primary (P), recurrences (R) | Immunocytochemistry | | | Southern blot analysis | | Interphase FISH | Cytogenetics |
|---|---|---|---|---|---|---|---|---|
| | | a | MDM2 | CDK4 | *MDM2* | *CDK4* | | |
| 1 | P | (2/7) | + | + | + | + | A | |
| 2 | P | (2/6) | + | + | + | + | | 48,XY,+r(12)×2/2n+,+der(12),+r(12),+d min |
| 3 | P | (2/9) | + | + | + | + | A | |
| 4 | R | (2/17) | + | + | + | + | A | |
| 5 | R | (2/13) | + | + | + | + | | |
| 6 | R | (2/26) | + | + | + | + | A | |
| 7 | P | (2/11) | + | + | + | + | A | 48,XY,+r(12)×2/2n+,+der(12),+r(12),+d min |
| 8 | R | (2/23) | + | + | + | + | | |
| 9 | P | (7/7) | – | + | + | + | | |
| 10 | R | (2/7) | + | + | | | | |
| 11 | R | (2/6) | + | + | | | | |
| 12 | R | (2/5) | + | + | | | | |
| 13 | R | (2/6) | + | + | | | | |
| 14 | P | (7/7) | – | + | | | | 48,XY,+2r |
| 15 | P | (2/26) | + | + | | | | |
| 16 | P | (8/8) | – | + | | | | |
| 17 | P | (2/8) | + | + | | | | |
| 18 | P | (2/4) | + | + | | | | |
| 19 | P | (2/4) | + | + | | | | |
| 20 | P | (2/9) | + | + | | | | |
| 21 | R | (2/5) | + | + | | | | |

[a]In brackets: number of samples immunostained for MDM2 and CDK4 by the total of specimens sampled for each tumour. A: features consistent with gene amplification.

**Table 3** Lipomas: immunophenotyping, molecular and cytogenetic features (19 cases)

| Case no. | Age gender | Site | size (cm) | Immunocytochemistry | | Southern blot analysis | | Interphase FISH | Cytogenetics |
|---|---|---|---|---|---|---|---|---|---|
| | | | | MDM2 | CDK4 | *MDM2* | *CDK4* | | |
| 22 | 54/F | Thigh | 18 | – | – | – | – | | |
| 23 | 79/M | Neck | 7 | – | – | – | – | | |
| 24 | 56/M | Lower back | 10 | – | – | – | – | TS | |
| 25 | 40/M | Thigh | 11 | – | – | – | – | TS | |
| 26 | 69/M | Arm | 7 | – | – | – | – | NE | 46,XY,add (12)(q13) |
| 27 | 67/M | Thorax region | 10 | – | – | – | – | | |
| 28 | 66/M | Abdominal wall | 30 | – | – | – | – | | |
| 29 | 39/M | Thorax region | 17 | – | – | | | | |
| 30 | 52/M | Thigh | 7 | – | – | | | | |
| 31 | 57/F | Abdominal wall | 13 | – | – | | | | |
| 32 | 66/M | Lower back | 13 | – | – | | | | |
| 33 | 76/M | Abdominal wall | 10 | – | – | | | | |
| 34 | 31/M | Thorax wall | 7 | – | – | | | | |
| 35 | 64/F | Shoulder | 9 | – | – | | | | |
| 36 | 59/M | Lower back | 11 | – | – | | | | |
| 37 | 68/M | Shoulder | 12 | – | – | | | | |
| 38 | 56/F | Thorax region | 7 | – | – | | | | |
| 39 | 53/F | Shoulder | 10 | – | – | | | | |
| 40 | 59/M | Thigh | 21 | – | – | | | TS | 46,XY t(6;18)(q13–14;q21) |

NE = not evaluable; TS = two signals.

immunophenotypic, genotypic and cytogenetic results. In keeping with immunophenotypic findings amplification of both *MDM2* and *CDK4* genes was observed in the nine LL-WDLs and no abnormality of the two genes was observed in the seven LDSLs analysed by Southern blot. Interestingly, case no. 9, categorized routinely on morphologic grounds as lipoma and reported as MDM2-negative in a previous investigation (Pilotti et al, 1997), showed CDK4 immunoreactivity (Figure 1C) and *CDK4* as well

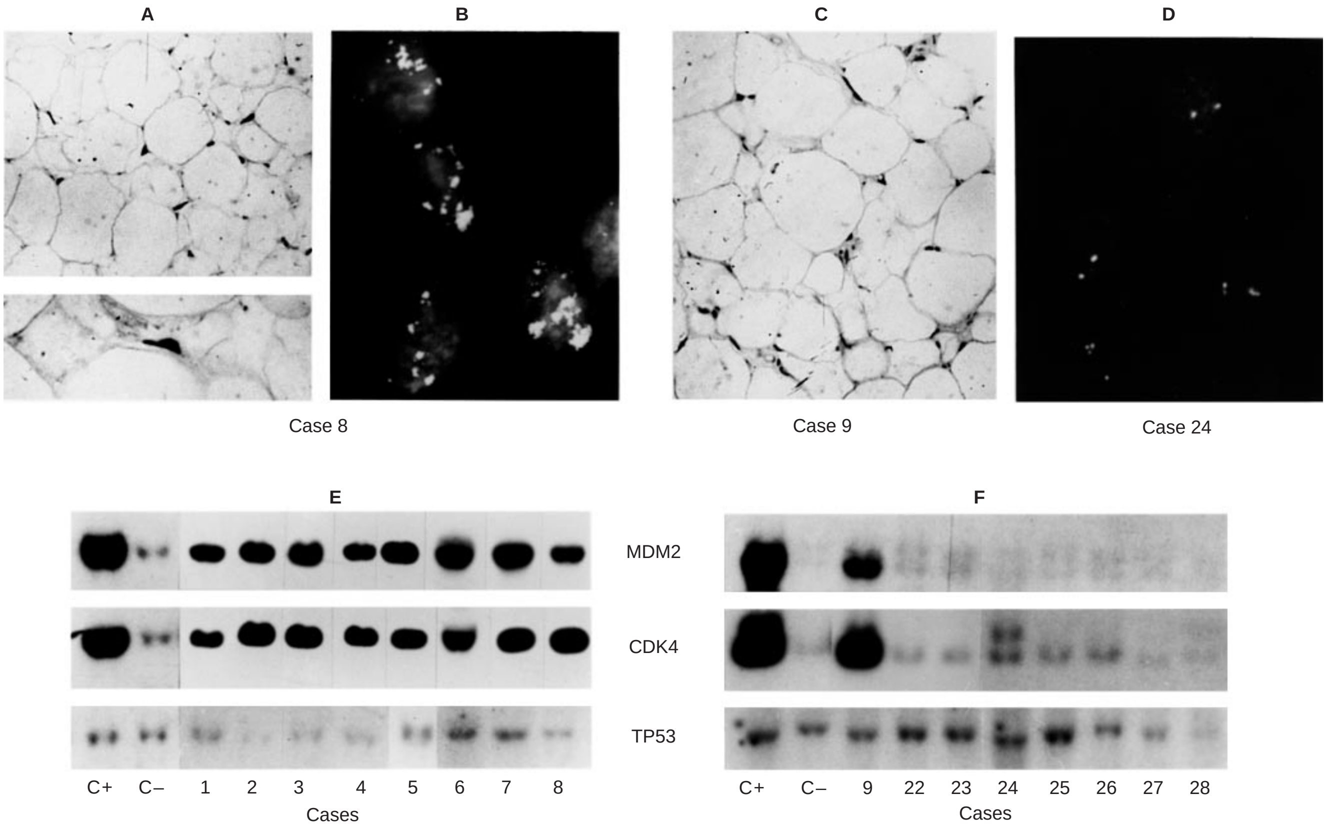

**Figure 1** (**A**) and inset: mdm2 expression, and (**B**) gene amplification by FISH, in one of the LL-WDL cases (case no. 8). (**C**) cdk4 immunoreactivity, in case no. 9 (previously classified as LDSL) null for mdm2 but showing coamplification at MDM2 and CDK4 genes by Southern blot (see below). (**D**) FISH showing two normal signals in one of the LDSL cases (case no. 24). (**E**) Southern blot analysis of MDM2 and CDK4 amplification in eight cases of LL-WDL. Hybridization with MDM2 or CDK4 probe reveals amplification of both genes in the tumour DNA of all cases. (**F**) Southern blot hybridization of 1 LL-WDL (previously classified as LDSL, case no. 9) and seven cases of LDSL. None of the LDSLs show amplification of MDM2 and CDK4 while the two genes are coamplified in the LL-WDL. As a control for the loading amount of DNA the blots in E and F were hybridized with a TP53 probe. C+: SJSA-1 osteosarcoma cell line positive for MDM2 and CDK4 amplification. C–: normal peripheral blood cells. The numbers on the bottom of the blots are in accordance with Tables 2 and 3

as *MDM2* gene amplification (Figure 1F) despite MDM2 null immunophenotype. In light of these findings the case, which since the time of the first categorization showed no completely convincing benign features, was reconsidered as possible LL-WDL and included in Table 1.

FISH was successfully applied in the five cases of LL-WDL and in three out of four cases of LDSL. Nuclei obtained from the LL-WDL cases showed an amplification level ranging from eight to 15 signals (Figure 1B). Although FISH cannot demonstrate that the amplified gene is *MDM2* (since the probe covered *MDM2*, *SAS* and *GLI* sequences), this assumption is supported by the immunostaining (Figure 1A) and Southern blot results obtained with the *MDM2*-specific probe (Figure 1E). By contrast, nuclei from LDSL showed two normal signals corresponding to the two copies of the region (1D). Karyotypic analysis showed the presence of ring chromosomes in three LL-WDL cases analysed; two of them (case no. 2 and no. 14) also showed the presence of a minor clone (10% of the total metaphases), that contained ring chromosomes, giant markers and double minutes. In these two cases FISH experiments with a whole chromosome 12 probe completely painted the ring chromosomes of the major clone, and the ring chromosomes, the double minutes and part of the giant markers of the minor clone. A translocation with a breakpoint at band q13 of chromosome 12 was found in case no. 26 of LDSL, and a novel translocation t(6;18)(q13–14; q21) was found in case no. 40 of LDSL.

### DISCUSSION

On cytogenetic grounds, about two-thirds of ordinary lipomas and most WDLs share aberrations in the chromosome 12q13–15 segment (Mandahl et al, 1996). However, in lipomas the majority of these aberrations are consistent with the creation of chimaeric genes made up of a fusion of the *HMG1-C* gene in 12q15 with multiple partners, although the LPP (lipoma preferred partner) gene, at 3q27–28, is preferentially involved (Petit et al, 1996). On the other hand, in WDLs the aberrations of chromosome 12q are represented by the amplification of genes spanning the 13q–15q region (Berner et al, 1997; Dei Tos and Dal Cin, 1997). Assuming that these findings represent two different transformation pathways, and taking into account that *MDM2* and *CDK4* gene amplification parallels the presence of ring chromosomes as well as MDM2 and CDK4 protein overexpression (Pilotti et al, 1997, 1998), it is reasonable to assume that immunophenotyping will assist in the differential diagnosis between LL-WDL and LDSL. The present data confirm this assumption that, in turn, makes immunophenotype more consistent with biology than morphology. Similar results have recently been observed using different, less easily handled and not always applicable methodologic approaches, such as classical cytogenetic methods and comparative genomic hybridization (CGH) (Suijkerbuijk et al, 1994; Mandahl et al, 1996; Szymanska et al, 1997).

 *© 2000 Cancer Research Campaign*

Support to our immunophenotypic results is provided by the molecular and cytogenetic data which fitted with the immunophenotypic information in all the examined cases. For the *MDM2* gene, the Southern blot data were further confirmed by FISH which showed amplification in the 5 LL-WDL analysed. By contrast, FISH revealed only two signals in the three LDSLs successfully analysed. Moreover, in one of these three cases of lipoma (case no. 26) the diagnosis was further supported by the presence of a translocation with a breakpoint at q13 of chromosome 12, a cytogenetic feature characteristic of lipoma (Heim and Mitelman, 1995), and a novel translocation t(6;18)(q13–14;q21) was found in a second case. Even though rearrangement of 12q13–14 and 6p are the most frequently found in lipoma, other observations which do not involve these chromosome arms have been reported (Willen et al, 1998). The finding that the MDM2 antibody failed to decorate tumour cells in two cases of LL-WDL (cases 14 and 16 respectively), points out the value of *CDK4* gene assessment in differential diagnosis, and reconfirms that even amplification excluding *MDM2* may contribute to transformation (Pilotti et al, 1998). It is rather unlikely, in fact, that immunostaining failure be ascribed to cellular variation as immunostaining was performed in all samples. However, this possibility cannot be completely excluded in case no. 9 because of the discrepancy between immunochemical and molecular analyses regarding the *MDM2* amplification which, on the other hand, could suggest that an *MDM2* gene amplification can occur without the expression of the relative product.

In conclusion our results support the notion that MDM2 and CDK4 immunophenotyping may represent a valuable ancillary procedure in the assessment of arguable LDSL cases. This diagnostic approach will also clarify if retroperitoneal lipoma represents an anecdotal occurrence or a reality (DeWeerd and Dockerty, 1952).

### ACKNOWLEDGEMENTS

This work was supported by grant from AIRC (Associazione Italiana per la Ricerca sul Cancro). We are grateful to Mario Azzini for professionally preparing the illustrations and to Alda Tosi for secretarial assistance.

### REFERENCES

Berner J-M, Meza-Zepeda LA, KoolsPFJ, Forus A, Schoenmakers EFPM, Van de Ven WJM, Fodstad Ø and Myklebost O (1997) HMGIC, the gene for an architectural transcription factor, is amplified and rearranged in a subset of human sarcomas. *Oncogene* **14**: 2935–2941

Dal Cin P, Kools P, Sciot R, De Wever I, Van Damme B, Van de Ven W and Van Den Berghe H (1993) Cytogenetic and fluorescence in situ hybridization investigation of ring chromosomes characterizing a specific pathologic subgroup of adipose tissue tumors. *Cancer Genet Cytogenet* **68**: 85–90

Dei Tos AP and Dal Cin P (1997) The role of cytogenetics in the classification of soft tissue tumours. *Virchows Arch* **431**: 83–94

DeWeerd JH and Dockerty MB (1952) Lipomatous retroperitoneal tumors. *Am J Surg* **84**: 397–407

Evans HL (1988) Liposarcoma and atypical lipomatous tumors: a study of 66 cases followed for a minimum of 10 years. *Surg Pathol* **1**: 41–54

Fletcher CDM, Akerman M, Dal Cin P, de Wever I, Mandhal N, Mertens F, Mitelman F, Rosai J, Rydholm A, Sciot R, Tallini G, van den Berghe H, van de Ven W, Vanni R and Willen H (1996) Correlation between clinicopathological features and karyotype in lipomatous tumors. A report of 178 cases from the chromosomes and morphology (CHAMP) collaborative study group. *Am J Pathol* **148**: 623–630

Forus A, Flørenes VA, Maelandsmo GM, Meltzer PS, Fodstad Ø and Myklebost O (1993) Mapping of amplification units in the q13–14 region of chromosome 12 in human sarcomas: some amplica do not include MDM2. *Cell Growth Differ* **4**: 1065–1070

Heim S and Mitelman F (1995) Cancer cytogenetics. Chromosomal and molecular genetic aberrations of tumor cells. 2nd edn, pp. 484–489. Wiley-Liss: New York

Hunter T and Pines J (1994) Cyclins and cancer. II: cyclin D and CDK inhibitors come of age. *Cell* **79**: 573–582

Khatib ZA, Matsushime H, Valentine M, Shapiro DN, Sherr CJ and Look AT (1993) Coamplification of the CDK4 gene with MDM2 and GLI in human sarcomas. *Cancer Res* **53**: 5535–5541

Leach FS, Tokino T, Meltzer P, Burrell M, Oliner JD, Smith S, Hill DE, Sidransky D, Kinzler KW and Vogelstein B (1993) p53 mutation and MDM2 amplification in human soft tissue sarcomas. *Cancer Res* **53**: 2231–2234

Lichter P, Chang Tang C, Call K, Hermanson G, Evans GA, Housman D and Ward DC (1990) High resolution mapping of human chromosome 11 by in situ hybridization with cosmid clones. *Science* **247**: 64–67

Mandahl N, Akerman M, Aman P, Dal Cin P, De Wever I, Fletcher CDM, Mertens F, Mitelman F, Rosai J, Rydholm A, Sciot R, Tallini G, van den Berghe H, Van de Ven W, Vanni R and Willen H (1996) Duplication of chromosome segment 12q15–24 is associated with atypical lipomatous tumors: a report of the CHAMP collaborative study group. *Int J Cancer* **67**: 632–635

Mentzel T and Fletcher CDM (1995) Lipomatous tumours of soft tissues: an update. *Virchows Arch* **427**: 353–363

Mezzelani A, Sozzi G, Pierotti MA and Pilotti S (1996) Rapid differential diagnosis of myxoid liposarcoma by fluorescence in situ hybridization on cytological preparations. *J Clin Pathol: Mol Pathol* **49**: M308–M309

Nilbert M, Rydholm A, Willen H, Mitelman F and Mandahl N (1994) MDM2 gene amplification correlates with ring chromosomes in soft tissue tumors. *Genes Chrom Cancer* **9**: 261–265

Pedeutour F, Suijkerbuijk RF, Van Gaal J, Van de Klundert W, Coindre J-M, Van Haelst A, Collin F, Huffermann K and Turc-Carel C (1993) Chromosome 12 origin in rings and giant markers in well-differentiated liposarcoma. *Cancer Genet Gytogenet* **66**: 133–134

Petit MMR, Mols R, Schoenmakers EF, Mandahl N and Van de Ven WJ (1996) LPP, the preferred fusion partner gene at HMGIC in lipomas, is a novel member of the LIM protein gene family. *Genomics* **36**: 118–129

Pilotti S, Della Torre G, Lavarino C, Di Palma S, Sozzi G, Minoletti F, Rao S, Pasquini G, Azzarelli A, Rilke F and Pierotti MA (1997) Distinct mdm2/p53 expression patterns in liposarcoma subgroups: implications for different pathogenetic mechanisms. *J Pathol* **181**: 14–24

Pilotti S, Della Torre G, Lavarino C, Sozzi G, Minoletti F, Vergani B, Azzarelli A, Rilke F and Pierotti MA (1998) Molecular abnormalities in liposarcoma: role of MDM2 and CDK4-containing amplicons at 12q13–22. *J Pathol* **185**: 188–190

Rosai J, Akerman M, Dal Cin P, DeWever I, Fletcher CDM, Mandhal N, Mertens F, Mitelman F, Rydholm A, Sciot R, Tallini G, Van Den Berghe H, Van De Ven W, Vanni R and Willen H (1996) Combined morphologic and karyotypic study of 59 atypical lipomatous tumors. Evaluation of their relationship and differential diagnosis with other adipose tissue tumors (a report of the CHAMP study group). *Am J Surg Pathol* **20**: 1182–1189

Schoenmakers EFPM, Wanschura S, Mols R, Bullerdiek J, Van den Berghe H and Van de Ven WJM (1995) Recurrent rearrangements in the high mobility group protein gene, HMGI-C, in benign mesenchymal tumors. *Nat Genet* **10**: 436–444

Smith SH, Weiss SW, Jankowski SA, Coccia MA and Meltzer PS (1992) SAS amplification in soft tissue sarcomas. *Cancer Res* **52**: 3746–3749

Suijkerbuijk RT, Olde Weghuis DEM, Van Den Berg M, Pedeuour F, Forus A, Myklebost O, Glier C, Turc-Carel C and Geurts van Kessel A (1994) Comparative genomic hybridization as a tool to define two distinct chromosome 12 amplification units in well-differentiated liposarcomas. *Genes Chromosomes Cancer* **9**: 292–295

Szymanska J, Virolainen M, Tarkkanen M, Wiklund T, Asko-Seljavaara S, Tukiainen E, Elomaa I, Blomqvist C and Knuutila S (1997) Overrepresentation of 1q21–23 and 12q13–21 in lipoma-like liposarcomas but not in benign lipomas: a comparative genomic hybridization study. *Cancer Genet Cytogenet* **99**: 14–18

Tkachenko A, Ashar HR, Meloni AM, Sandberg AA and Chada KK (1997) Misexpression of disrupted HMGI architectural factors activates pathways of tumorigenesis. *Cancer Res* **57**: 2276–2280

Willen H, Akerman M, Dal Cin P, De Wever I, Fletcher CDM, Mandahl N, Mertens F, Mitelman F, Rosai J, Rydholm A, Sciot R, Tallini G, Van Den Berghe H and Vanni R (1998) Comparison of chromosomal patterns with clinical features in 165 lipomas: a report of the CHAMP study group. *Cancer Genet Cytogenet* **102**: 46–49

Zauberman A, Flusberg D, Haupt Y, Barak Y and Oren M (1995) A functional p53-responsive intronic promoter is contained within the human mdm2 gene. *Nucleic Acids Res* **23**: 2584–2592

© 2000 Cancer Research Campaign 
